# Reference values for bone metabolism in a Japanese cohort survey randomly sampled from a basic elderly resident registry

**DOI:** 10.1038/s41598-021-87393-7

**Published:** 2021-04-09

**Authors:** Ryosuke Tokida, Masashi Uehara, Masaki Nakano, Takako Suzuki, Noriko Sakai, Shota Ikegami, Jun Takahashi, Yukio Nakamura, Hiroyuki Kato

**Affiliations:** 1 Department of Orthopaedic Surgery, Shinshu University School of Medicine, 3-1-1 Asahi, Matsumoto, Nagano 390-8621 Japan; 2 Department of Orthopaedic Surgery, Nagano Children’s Hospital, 3100 Toyoshina, Azumino, Nagano 399-8288 Japan; 3 Department of Human Nutrition, Faculty of Human Nutrition, Tokyo Kasei Gakuin University, 22 Sanban-cho, Chiyoda-ku, Tokyo, 102-8341 Japan

**Keywords:** Endocrinology, Health care

## Abstract

The aim of this study was to provide definitive reference values for bone mineral density (BMD) and bone turnover markers in the general elderly population. Registered citizens of 50 to 89 years old were targeted for this survey. After random sampling from the resident registry of Obuse town, we established eight groups based on age (50 s, 60 s, 70 s, and 80 s) and gender. A total of 411 people were enrolled. We used a dual-energy x-ray absorptiometry device to measure and evaluate BMD. The bone formation marker bone alkaline phosphatase (BAP) was measured as a bone turnover marker. Bone quality marker pentosidine, and bone resorption markers including urinary total deoxypyridinoline (DPD), tartrate-resistant acid phosphatase 5b (TRACP-5b), 25-hydroxyvitamin D (25[OH]D), and whole parathyroid hormone (PTH) were also measured as bone turnover markers. Sixty-three people (15.3%) were diagnosed as osteoporosis. BMD decreased with age in the femoral neck and total hip. On the other hand, there was no characteristic change with age in the lumber spine. As for bone markers, pentosidine and DPD increased with aging, although 25(OH)D, whole PTH, and BAP showed no characteristic associations with gender and aging. In terms of the relationship between low BMD and bone markers, there was a significant independent association between low BMD and TRACP-5b in females. In conclusions, hip BMD decreased with aging in men and women. However, there was no characteristic decline with aging in the lumbar spine. All bone markers showed no significant independent characteristics associated with age or gender in a multivariate analysis model, except for a significant association between low BMD and TRACP-5b in females. TRACP-5b was a potentially useful marker for the detection of low BMD.

## Introduction

Bone mineral density (BMD) and bone turnover markers have become widely adopted as evaluation tools for osteoporotic disease^1–3^. There have been several reports on age-specific changes in BMD and bone turnover markers between men and women^4–8^, and clarification of age- and gender-specific reference is useful in osteoporosis treatment. Although several reports have attempted to determine reference values for BMD and bone turnover markers^9–12^, the availability of definitive reference values for BMD and bone turnover markers in the general population are scarce, since earlier studies were based on volunteer cohorts or lumbar disorder patients.

To establish a new population study of the Japanese subjects, we conducted a random sampling from the Obuse Town Registry of Residents to obtain a more representative cohort of the general population with minimal selection bias^13–16^. This epidemiological study is referred to as the "Obuse Study" after the name of the cooperating municipality of Obuse Town. It is the first study of its kind to provide baseline values for age-specific bone turnover markers in a large cohort study.

The present investigation proposes reference values for BMD and bone turnover markers in the Japanese population using the Obuse study cohort.

## Materials and methods

### Subjects

**Figure 1:**
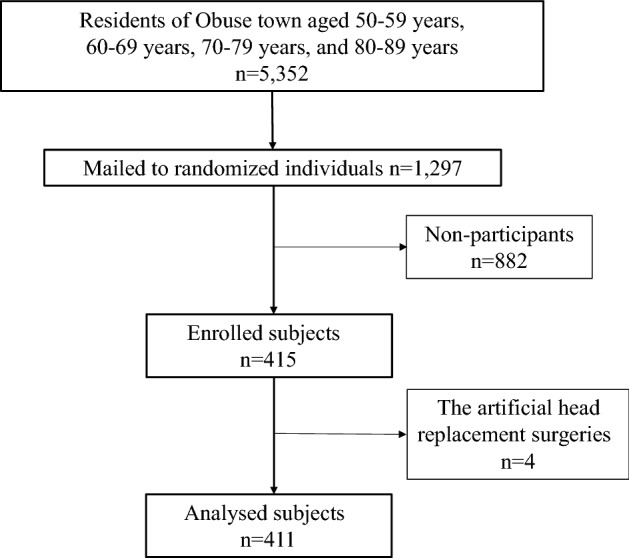
Obuse town resident participant flowchart. 1297 people were randomly sampled from 5352 residents aged between 50 and 89 years in the basic resident registry of Obuse town. A total of 415 people joined the Obuse study cohort, but 4 were excluded due to the artificial head replacement surgeries.

The protocols in this study conformed to the ethical guidelines of the 2013 Declaration of Helsinki and STROBE statement. Informed consent was obtained from participants prior to the initiation of the study. Participants were informed about the purposes of the research both verbally and in writing prior to the study, and written informed consent was obtained from all participants. This study was approved by the Institutional Review Board of Shinshu University (study no: 2792). From October 2014 to June 2017, we conducted an epidemiological study of residents (the Obuse Study) as a joint collaboration with a cooperating town office^13, 14^. Male and female participants between the ages of 50–89 were randomly selected from a pool of 5,352 registrants in the resident registry of a rural town^13, 14^. Those selected from the registry were asked whether they would be able to undergo a bone density examination, and calls for participation were continued until approximately 50 consenting participants were successfully recruited for each age group and sex^13, 14^. Four hundred and eleven participants were consequently included in the study, excluding 4 participants with incomplete measurements (Fig. 1)^13, 14^.

### Bone mineral density and young adult mean measurement

We used a dual-energy x-ray absorptiometry device (GE Prodigy, GE healthcare, Chicago, IL, USA) to measure and evaluate bone mineral density (BMD) and T-score. BMD and T-score were measured at the femoral neck, total hip, and lumbar spine (L2–4). Based on the WHO diagnostic criteria, T-score ≥ − 1 was classified as healthy, − 2.5 < T-score < − 1 as osteopenia, and T-score ≤ − 2.5 as osteoporosis^17^. Osteoporosis and osteopenia were defined as low BMD.

### Assay of bone turnover markers

The bone quality marker pentosidine, as well as the bone resorption marker urinary total deoxypyridinoline (DPD), tartrate-resistant acid phosphatase 5b (TRACP-5b), 25-hydroxyvitamin D (25[OH]D), and whole parathyroid hormone (PTH) were measured as bone turnover markers. Serum pentosidine, TRACP-5b, 25(OH)D, and whole PTH were measured using an enzyme-linked immunosorbent assay kit (SRL, Tokyo, Japan), enzyme immunoassay kit (SRL), electro chemiluminescence immunoassay kit (SRL), and chemiluminescent enzyme immunoassay kit (SRL), respectively. Urine DPD was measured using an enzyme immunoassay kit (SRL). The bone formation marker bone alkaline phosphatase (BAP) was also measured as a bone turnover marker. Serum BAP was measured using a chemiluminescent enzyme immunoassay kit (SRL).

### Statistical analysis

The prevalence of osteoporosis was compared for each age group and sex. BMD and bone turnover markers were evaluated for each age and sex using Tukey’s test for comparisons among multiple groups. Multiple logistic regression analysis was applied to determine the association between bone markers and low BMD.

All statistical analyses were performed with EZR (Saitama Medical Center, Jichi Medical University), which is a graphical user interface for R software (version 3.5.2; The R Foundation for Statistical Computing, Vienna, Austria).

## Results

Of the 415 participants who were randomly sampled from the resident registry, 4 participants who were unable to measure BMD due to artificial hip joint replacement were excluded (Fig. 1). The physical characteristics and functions of the 411 examinees are shown for each age group and sex in Table 1. The interview results regarding participant comorbidities and menopause were shown in Table 2. Thirty-nine (9.5%) people had been treated for osteoporosis. Fifteen (3.6%) people had been treated with hormone therapy. Five (1.2%) people had been treated with steroid. A history of vertebral fracture was observed in 15 subjects (3.6%).

**Table 1:** Characteristics of 411 subjects in the Obuse study cohort.

	Age strata (years)	n	Height (cm)	Weight (kg)	BMI (kg/m2)
Male	50–59	50	171.8 (5.9)	67.1 (9.0)	22.7 (2.9)
	60–69	53	166.7 (4.7)	66.9 (7.7)	24.1 (2.7)
	70–79	55	163.2 (4.9)	60.0 (10.2)	22.5 (3.4)
	80–89	45	160.1 (5.6)	57.5 (8.4)	22.4 (2.7)
	Total	203	165.6 (6.8)	63.0 (0.98)	22.9 (0.30)
Female	50–59	47	158.1 (4.9)	55.4 (8.9)	22.2 (3.8)
	60–69	61	152.8 (5.3)	52.2 (7.6)	22.3 (2.7)
	70–79	55	149.3 (5.6)	50.7 (7.9)	22.8 (3.5)
	80–89	45	144.7 (6.1)	48.4 (8.1)	23.1 (3.4)
	Total	208	151.3 (7.2)	51.7 (8.4)	22.6 (3.3)

Values represent mean (standard deviation).

BMI, Body Mass Index.

**Table 2:** Comorbidities in the study cohort.

Disease	No. of participants	Prevalence (%)
Hyperthyroidism	5	1.2
Hyperparathyroidism	0	0
Diabetes mellitus	52	12.7
Paget's disease of bone	3	0.7
Rheumatoid arthritis	5	1.2
Chronic obstructive pulmonary disease	7	1.7
Fracture	131	31.9
Menopause (only female)	202	96.7

Sixty-three people (15.3%) were diagnosed as osteoporosis, of which 14 (6.9%) were male and 49 (23.6%) were female. In men and women, diminished hip BMD was seen in the elderly. The decrease in BMD was particularly pronounced in the femoral neck. On the other hand, there was no characteristic change with age in the lumber spine (Table 3).

**Table 3:** Bone mineral density and T-score at the femoral neck, proximal femur, and lumbar 1–4, in addition to prevalence of osteoporosis.

	Age strata (years)	N	Femoral neck BMD	Total hip BMD	Lumbar spine BMD	Lumbar spine BMD without vertebral fracture	Femoral neck T-score	Total hip T-score	Lumbar spine T-score	Number of OP
Male	50–59	50	0.90 (0.13)	0.96 (0.13)	1.17 (0.18)	1.17 (0.18)	− 0.39 (1.04)	0.13 (1.01)	− 0.13 (1.53)	2 (4.0%)
	60–69	53	0.91 (0.11)	0.99 (0.13)	1.28 (0.22)	1.28 (0.22)	− 0.35 (0.88)	0.35 (0.99)	0.76 (1.84)	0 (0.0%)
	70–79	55	0.88 (0.12)	0.97 (0.15)	1.35 (0.27)^a^	1.36 (0.27)^a^	− 0.51 (0.93)	0.21 (1.14)	1.31 (2.28)	5 (9.1%)
	80–89	45	0.80 (0.15)^abc^	0.87 (0.15)^abc^	1.27 (0.29)	1.28 (0.29)	− 1.12 (1.12)	− 0.59 (1.14)	0.70 (2.39)	7 (15.6%)
	Total	203	0.88 (0.13)	0.95 (0.15)	1.27 (0.25)	1.27 (0.25)	− 0.58 (1.03)	0.05 (1.12)	0.67 (2.08)	14 (6.9%)
Female	50–59	47	0.80 (0.11)	0.87 (0.13)	1.09 (0.18)	1.09 (0.18)	− 0.85 (0.90)	− 0.52 (1.06)	− 0.24 (1.46)	4 (8.5%)
	60–69	61	0.76 (0.10)	0.84 (0.11)	1.02 (0.18)	1.02 (0.18)	− 1.16 (0.85)	− 0.81 (0.88)	− 0.80 (1.52)	12 (19.7%)
	70–79	55	0.72 (0.10)^a^	0.80 (0.12)^a^	1.03 (0.20)	1.02 (0.21)	− 1.47 (0.86)	− 1.08 (0.99)	− 0.80 (1.67)	15 (27.3%)
	80–89	45	0.67 (0.10)^ab^	0.71 (0.10)^abc^	1.00 (0.20)	1.00 (0.20)	− 1.88 (0.80)	− 1.83 (0.84)	− 0.96 (1.67)	18 (40.0%)
	Total	208	0.74 (0.11)	0.81 (0.13)	1.04 (0.19)	1.03 (0.19)	− 1.33 (0.92)	− 1.04 (1.05)	− 0.71 (1.60)	49 (23.6%)

Values represent mean (standard deviation).

Values of OP represent number (prevalence).

One female patient aged 70s and 3 female patients aged 80s were excluded due to the artificial head replacement surgeries.

BMD: bone mineral density, OP: osteoporosis.

^a^Significantly different (p < 0.05) values from those aged 50–59 years.

^b^Significantly different (p < 0.05) values from those aged 60–69 years.

^c^Significantly different (p < 0.05) values from those aged 70–79 years.

In men and women, pentosidine and DPD increased with aging. In addition, TRACP-5b increased with age in males. 25(OH)D, whole PTH, and BAP showed no characteristics associated with gender or aging (Table 4). In addition, bone turnover markers, pentosidine, and whole PTH were compared in the presence and absence of OP. Pentosidine was 0.062 ± 0.022 in OP and 0.054 ± 0.025 in non-OP, which was significantly greater in OP (p = 0.028). DPD was 32.2 ± 25.7 in OP and 34.0 ± 25.0 in non-OP, with no significant difference between the two groups (p = 0.64). BAP was 15.9 ± 5.8 in OP and 14.0 ± 4.4 in non-OP, and which was significantly greater in OP (p = 0.030). TRACP-5b was 505 ± 198 in OP and 422 ± 160 in non-OP, significantly greater in OP (p = 0.007). Whole PTH was 24.0 ± 14.7 in OP and 20.7 ± 8.3 in non-OP, and there was no significant difference between the two groups (p = 0.128). 25(OH)D was 21.4 ± 7.6 in OP and 23.7 ± 7.2 in non-OP, with no significant difference between the two groups (p = 0.055).

**Table 4:** Bone markers and 25-hydroxyvitamin D.

	Age strata (years)	Pentosidine	DPD	25(OH)D	TRACP-5b	Whole PTH	BAP
Male	50–59	0.05 (0.01)	3.3 (0.8)	25.2 (6.0)	312.2 (88.3)	21.8 (8.1)	11.9 (2.4)
	60–69	0.05 (0.02)	3.7 (1.2)	22.9 (5.4)	380.8 (144.0)	19.8 (7.7)	13.5 (3.8)
	70–79	0.06 (0.02)^a^	3.9 (1.1)^a^	29.3 (7.5)^ab^	448.7 (198.4)^a^	20.7 (7.7)	13.6 (4.7)
	80–89	0.07 (0.02)^abc^	5.2 (1.7)^abc^	22.0 (5.6)^c^	489.7 (194.8)^ab^	22.0 (12.1)	13.3 (3.7)
	Total	0.06 (0.02)	4.0 (1.4)	25.0 (6.8)	406.2 (174.2)	21.0 (8.9)	13.1 (3.8)
Female	50–59	0.04 (0.01)	5.9 (1.2)	22.0 (6.5)	416.1(130.9)	22.6 (11.0)	15.2 (4.5)
	60–69	0.05 (0.02)	5.3 (1.3)	20.6 (6.2)	478.5 (141.7)	20.1 (7.7)	16.1 (5.1)
	70–79	0.06 (0.05)^a^	5.4 (2.0)	25.0 (9.1)^b^	490.1 (167.4)	22.2 (12.3)	15.5 (5.4)
	80–89	0.06 (0.02)^a^	6.2 (2.5)^b^	19.2 (6.0)^c^	433.8 (167.9)	21.0 (10.5)	14.1 (5.3)
	Total	0.06 (0.03)	5.7 (1.8)	21.8 (7.4)	457.6 (154.8)	21.4 (10.4)	15.3 (5.1)

Values represent mean (standard deviation).

DPD: deoxypyridinoline, 25(OH)D: 25-hydroxyvitamin D, TRACP-5b: tartrate-resistant acid phosphatase 5b, PTH: parathyroid hormone, BAP: bone alkaline phosphatase.

^a^Significantly different (p < 0.05) values from those aged 50–59 years.

^b^Significantly different (p < 0.05) values from those aged 60–69 years.

^c^Significantly different (p < 0.05) values from those aged 70–79 years.

Relevant factors selected by the unifactorial analysis were subjected to multiple logistic regression analysis with gender. The results showed that when considering low BMD as a dependent variable, bone markers were not associated significantly with low BMD in males (Table 5). However, there was a significant independent association between low BMD and TRACP-5b in females (Table 6).

**Table 5:** Independent association between low BMD and bone markers in males.

Factor	Univariate		Multivariate	
	Odds ratio (95%CI)	*p* value	Odds ratio (95%CI)	*p* value
Age	1.20 (0.91–1.60)	0.20		
Pentosidine	1.11 (0.84–1.47)	0.47		
DPD	1.40 (1.04–1.87)	0.026	1.18 (0.84–1.67)	0.34
25(OH)D	0.86 (0.65–1.14)	0.30		
TRACP-5b	1.51 (1.11–2.05)	0.0079	1.38 (0.97–1.97)	0.072
Who1e PTH	1.32 (0.98–1.77)	0.064		
BAP	1.08 (0.82–1.41)	0.60		

CI: confidence interval, DPD: deoxypyridinoline, 25(OH)D: 25-hydroxyvitamin D, TRACP-5b: tartrate-resistant acid phosphatase 5b, PTH: parathyroid hormone, BAP: bone alkaline phosphatase.

**Table 6:** Independent association between low BMD and bone markers in females.

Factor	Univariate		Multivariate	
	Odds ratio (95%CI)	*p* value	Odds ratio (95%CI)	*p* value
Age	2.23 (1.54–3.21)	0.00002	1.90 (1.28–2.83)	0.0015
Pentosidine	1.94 (1.01–3.70)	0.045	1.36 (0.70–2.66)	0.366
DPD	1.35 (0.93–1.95)	1.95		
25(OH)D	0.74 (0.54–0.99)	0.048	0.74 (0.53–1.03)	0.075
TRACP-5b	1.56 (1.11–2.20)	0.011	1.57 (1.07–2.31)	0.021
Who1e PTH	1.26 (0.85–1.85)	0.25		
BAP	1.25 (0.90–1.75)	0.19		

CI: confidence interval, DPD: deoxypyridinoline, 25(OH)D: 25-hydroxyvitamin D, TRACP-5b: tartrate-resistant acid phosphatase 5b, PTH: parathyroid hormone, BAP: bone alkaline phosphatase.

## Discussion

In the present cohort study, we were able to calculate mean BMD and bone turnover markers by age and sex for the elderly aged 50 years and older according to the Japanese population ratio in more than 400 subjects randomly selected from a rural town registry in Japan. We were able to create a cohort that more accurately reflects the general population in comparison to traditional population studies that recruited active volunteers. Another feature of this study was the uniform distribution of age and gender ratios between 50 and 89 years old, as a result of collecting about 50 physical examination participants by age and gender. This uniform distribution is advantageous for making accurate statistical comparisons between men and women and between age groups.

In this study, significantly diminished BMD was seen in the elderly at the femoral neck and total hip in males and females. The BMD of the femoral neck, total hip, and lumber spine were comparable with previous studies in our country and elsewhere after accounting for gender and age (Table 7). Previous studies have demonstrated that BMD decreased with aging^18, 19^, and this study obtained the same results as have been described in the literature.

**Table 7:** Mean BMD values in previous reports.

Study	Country	Sex	Number	Age (years)	Femoral neck	Total hip	Lumber spine
Lee, 2019	Korea	Male	244	Age > 65	0.78 (0.007)	0.87 (0.008)	0.94 (0.005)
		Female	319		0.56 (0.005)	0.70 (0.005)	0.73 (0.008)
Schacht, 2019	Denmark	Male	98	69.0 (6.0)	0.95 (0.18)	1.10 (0.24)	1.31 (0.26)
		Female	86	70.0 (5.8)	0.83 (0.19)	0.88 (0.24)	1.13 (0.25)
Fuggle, 2018	England and Wales	Male	194	64.4 (2.5)	1.03 (0.14)		1.06 (0.15)
		Female	171	66.5 (2.7)	0.89 (0.13)		0.95 (0.17)
Fujiwara, 2003	Japan	Male	763	62.9 (9.8)	0.73 (0.11)		0.98 (0.16)
		Female	1593	65.4 (9.8)	0.62 (0.11)		0.82 (0.11)
Our study	Japan	Male	203	69.5 (11.2)	0.88 (0.13)	0.95 (0.15)	1.27 (0.25)
		Female	208	70.0 (11.0)	0.74 (0.11)	0.81 (0.13)	1.04 (0.19)

Values represent mean (standard deviation).

Vertebral fracture should be considered as an influence on lumbar BMD, but vertebral fracture in this study was only 3.6%, and we do not think it has a significant influence on the results of lumbar BMD. We also performed an analysis of lumbar BMD without the subject of vertebral fractures, and the values were similar (Table 3). All bone markers in this study were within the standard value for males and females in each generation^10, 12^. In men and women, pentosidine and DPD increased with age. TRACP-5b increased with aging in males. However, other markers showed no association with age. In the current study, subjects were randomly selected from a rural Japanese town; thus, subjects may be healthy. Aging may exert little influence on bone markers, while the prevalence of osteoporosis and osteopenia increased with aging in both males and females.

In terms of the association between low BMD and bone markers, there was a significant association between low BMD and TRACP-5b in females. TRACP-5b is a bone resorption marker that is not affected by renal dysfunction and has a low diurnal variability^20, 21^. Thus, TRACP-5b has been considered a useful marker. TRACP-5b was inversely correlated with BMD in females^22^. Furthermore, TRACP-5b has been described to be associated with increased fracture risk in elderly females^23, 24^. TRACP-5b could be a potential marker to predict fractures. In this study, TRACP-5b was related to low BMD in randomly selected female residents in the area. High bone resorption may be a factor for low BMD in female residents. TRACP-5b may be a marker which was useful for the detection of low BMD.

Several reports have shown that diabetic patients have a higher risk of fractures, even though they do not have decreased BMD^25–29^. On the other hand, pentosidine has been found to increase in DM patients^30^. Pentosidine, one of the advanced glycation end products, is a marker of bone quality (matrix) and is associated with fragility fractures independently of bone density^31^, and can be associated with the pathogenesis of bone fragility in patients with DM. The most common comorbidity in this cohort was DM. There may have been some effect on the pentosidine levels. However, since the purpose of our study was to present data from a population close to the general population, not volunteers, we believe it is meaningful to present data including subjects with DM.

For females, previous studies reported large differences between pre- and postmenopausal serum bone turnover markers concentrations^32, 33^. During the menopausal transition, bone turnover markers increase due to increased osteoclast activity, which is caused by decreased estrogen levels^34^. In terms of BMD, it was reported that the degree of decline with age was greater in women than in men^4, 6^. On the other hand, Hannan et al. reported that men have higher bone mineral density than women, but there is no difference in the degree of bone mineral density loss with age^6^.

Bone turnover markers levels are high in early puberty and then decline faster in girls than in boys^35–37^. In young adults, bone turnover markers levels are higher in males than in females and then decline faster in females, reaching their lowest levels in the 40 s in females and in the 50 s in males^38^. PINP and CTX-I increase at menopause and then remain higher than premenopausal levels^39, 40^. In contrast, in older men, CTX-I and PINP levels are generally stable or only slightly elevated past age 70^41^.

There is a limitation in this study. Although the research design reduces the sampling bias by adopting random sampling from the resident register, we may not have been able to control for all potential biases as a result of the low participation rate.

## Conclusions

A characteristic feature of this study was the collection of participants with age ranging from 50 to 89 years by a randomly sampling from the resident register.

Therefore, this research was designed to create a cohort that more accurately reflects common residents. BMD decreased with aging in the femoral neck and total hip. On the other hand, there was no characteristic change with aging in the lumber spine. Furthermore, all bone markers except for pentosidine and DPD showed no significant independent characteristics associated with age or gender in a multivariate analysis model. However, a significant association between low BMD and TRACP-5b in females was observed; therefore, high bone resorption may be a factor for low BMD in female residents.
